# Supplementary material for: Application of Pluronics for Enhancing Aqueous Solubility of Lipophilic Microtubule Destabilizing Compounds on the Sea Urchin Embryo Model
Source: Int J Mol Sci. 2023 Sep 28;24(19):14695. doi: 10.3390/ijms241914695 (PMC10572563; doi:10.3390/ijms241914695)
Supplement: Supplementary file 1 [file ijms-24-14695-s001.zip › ijms-2604165-supplementary.pdf]

Supplementary Data

Application of Pluronics for Enhancing Aqueous Solubility of Lipophilic Microtubule Destabilizing Compounds on the Sea Urchin Embryo Model

Marina N. Semenova [1], Nikolay S. Melik-Nubarov [2], Victor V. Semenov[3,]*

[1] *N. K. Koltzov Institute of Developmental Biology RAS, 26 Vavilov Street, 119334 Moscow, Russian Federation*

[2] *Department of Chemistry, M.V. Lomonosov Moscow State University, Leninskie Gory, 1/11B, Moscow, 119991, Russian Federation*

[3] *N. D. Zelinsky Institute of Organic Chemistry RAS, 47 Leninsky Prospect, 119991 Moscow, Russian Federation*

* Corresponding author: Prof. Victor V. Semenov at N. D. Zelinsky Institute of Organic Chemistry RAS, 47 Leninsky Prospect, 119991 Moscow, Russian Federation; tel: +7 (916) 620-9584; fax: +7 (499) 137-2966; E-mail: vs@zelinsky.ru

Table of content

Figure S1. UV spectra of chalcones and their absorbance……………………………….……………Page S2

Figure S2. Sea urchin embryo developmental stages selected for recording Pluronics effects….Page S3

Table S1. Morphological effects of Pluronic F127 on sea urchin embryos……………………..Page S4

Table S2. Effects of Pluronic P123 on sea urchin embryos……………………………………..Page S5

Table S3. Morphological effects of Pluronic L121 on sea urchin embryos……………………..Page S6

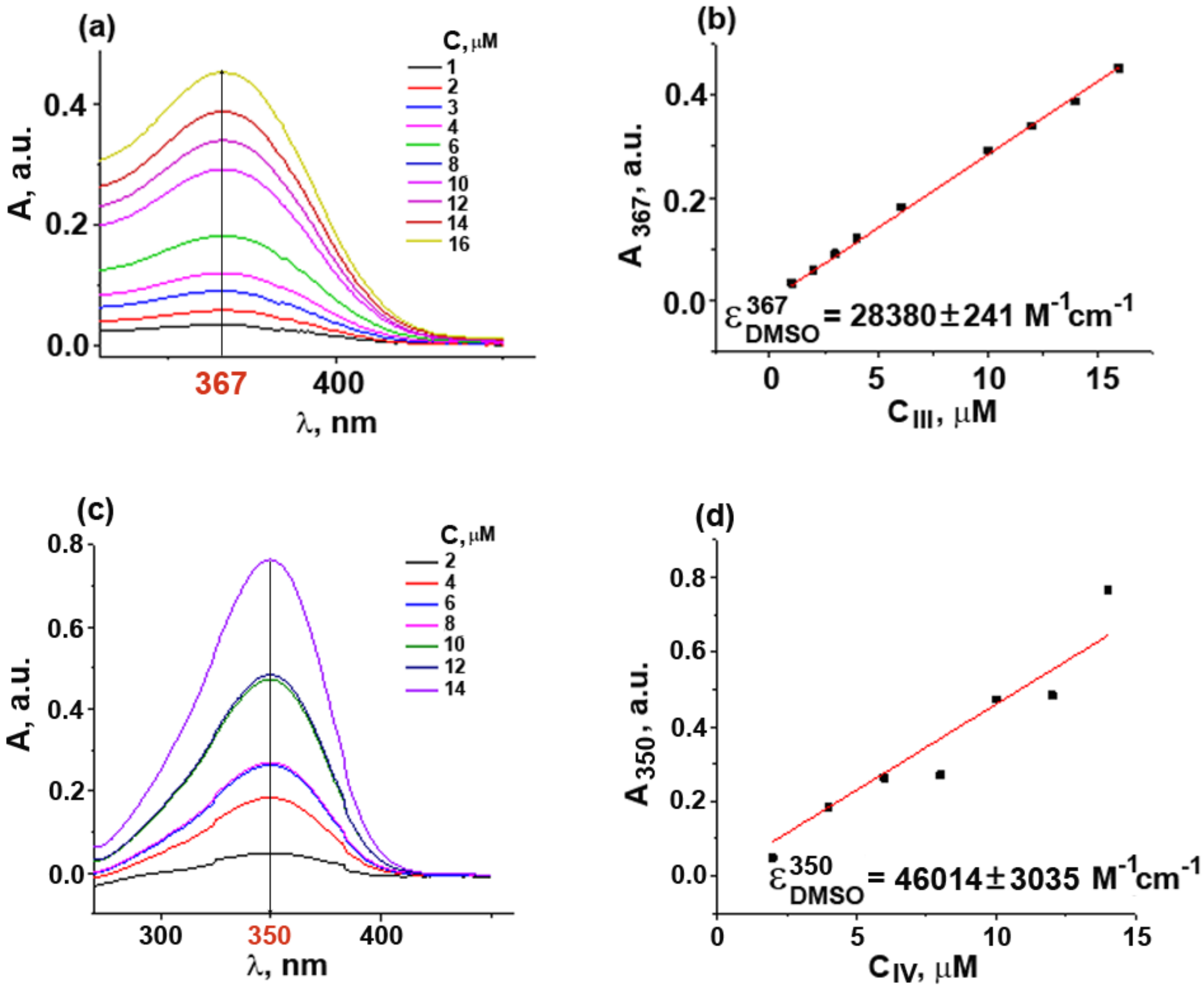

Figure S1. Determination of molar extinction coefficients (ε_{DMSO}) of chalcones **3** and **4**. Concentration-absorbance graphs of the standard 1–16 μM DMSO solutions of chalcones **3** (a) and **4** (c) and their absorbance (A) at 367 nm (b) and 350 nm (d), respectively, as a function of concentration. The confidence intervals of ε_{DMSO} values were calculated from least squares analysis.

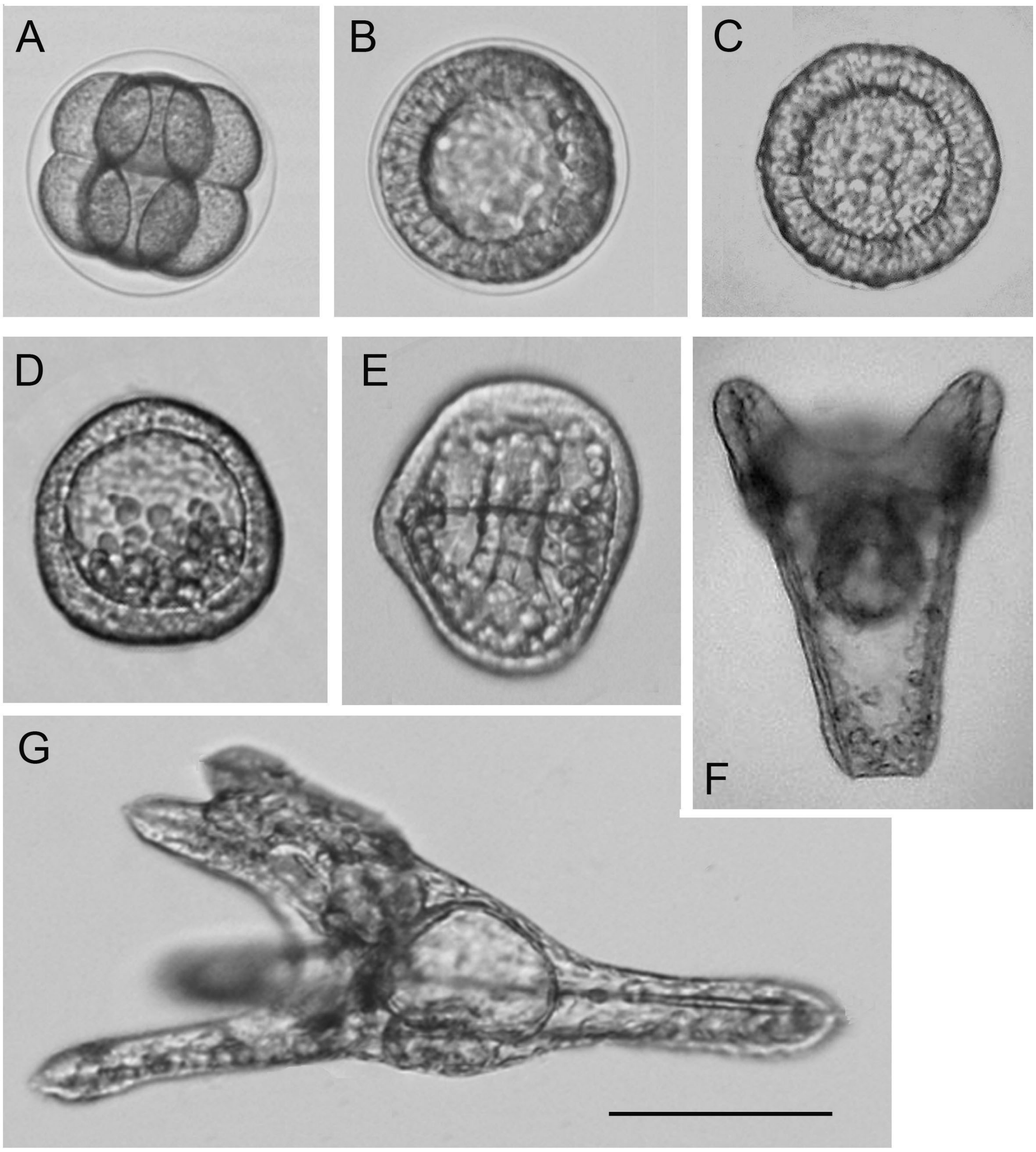

Figure S2. Sea urchin embryo developmental stages selected for recording Pluronics effects. (A) Eight cell embryo, 2.5 h. (B) Early blastula, 5.5 h. (C) Hatched mid-blastula, 8.5 h. (D) Mesenchyme blastula, 11 h. (E) Prism, 20 h. (F) Two-arm early pluteus, 26 h. (G) Four-arm mid-pluteus, 34 h. Incubation temperature: 24 °C. Scale bar: 100 µM.

Table S1. Morphological effects of Pluronic F127 on sea urchin embryos.

Concentration		Effect[a]
% w/v	µM	
0.05	39.7	**NOEC.** No effect.
0.1	79	**MEC.** Fertilization envelope softening in a part of eggs/embryos resulting in embryo deformation without developmental defects. Embryo deformation was observed up to late gastrula stage. No difference from intact embryos was detected at prism and pluteus stages.
0.2	158	Fertilization envelope softening resulting in embryo deformation without developmental defects. Embryo deformation was observed up to prism stage. Later, no difference from intact plutei was detected.
0.4	316	Fertilization envelope softening resulting in moderate embryo deformation at blastula - early pluteus stage. Hatching and swimming were not affected.
0.8	635	Fertilization envelope softening resulting in pronounced embryo deformation, formation of lentil-like shape blastules that further developed into plutei with flattened apex. Minor plutei growth inhibition. Hatching and swimming were not affected.
1.5	1190	Fertilization envelope softening resulting in pronounced embryo deformation, formation of lentil-like shape blastules that further developed into malformed plutei. Hatching and swimming were not affected.
2.5	1985	Fertilization envelope softening resulting in pronounced embryo deformation, formation of lentil-like shape blastules that further developed into slowly swimmimg dark flattened gastrules. Hatching was not affected.
5	3970	**MLC.** Strong embryo deformation, lentil-like embryo shape, embryo death after 9.5 h of treatment, when control embryos reached hatched blastula stage.

[a] Morphological effects were concentration-related and independent of room temperature within the interval of 21–25 °C. Duplicate measurements showed no differences in NOEC, MEC, and MLC values.

Table S2. Effects of Pluronic P123 on sea urchin embryos.

Mean T, °C	NOEC		MEC (minor effect)[a]		EC (strong effect)[b]		MLC	
	% w/v	μM	% w/v	μM	% w/v	μM	% w/v	μM
24	0.005	8.6	0.01–0.02	17.25–34.5	0.05	86	0.1	172.5
23	0.005	8.6	0.01–0.02	17.25–34.5	0.02	34.5	0.05	86
21	0.00125	2.15	0.0025–0.005	4.3–8.6	0.005–0.01	8.6–17.25	0.01–0.02	17.25–34.5
16	0.00125	2.15	0.0025[c]	4.3[c]	0.0025[c]	4.3[c]	0.005	8.6

[a] Minor effect: Minor developmental delay from cleavage stage; minor plutei growth inhibition.

[b] Strong effect: Pronounced cleavage inhibition with further hatching delay and post-hatching developmental retardation. Formation of early plutei without arms.

[c] At 16 °C MEC of P123 caused strong effect.

Table S3. Morphological effects of Pluronic L121 on sea urchin embryos.

**Concentration**[a]		**Effect**[b]
**% w/v**	**μM**	
0.000625	1.42	**NOEC.** No effect.
0.00125	2.84	**MEC.** Minor plutei growth inhibition. Cleavage, hatching, and swimming were not affected.
0.0025	5.7	Minor developmental delay from cleavage stage; plutei growth inhibition. Swimming was not affected.
0.005	11.4	Strong cleavage inhibition, hatching delay and post-hatching developmental retardation. Formation of early plutei without arms. Swimming was not affected.
0.01	22.7	**MLC.** Cleavage arrest at 2–8-cell and morula stages followed by embryo death after ~10 h of treatment.
0.02	45.4	Cleavage arrest and formation of 2–4-cell embryos followed by embryo death after ~9 h of treatment.
0.05–0.2	114–454	No cleavage. Destruction of arrested eggs after ~ 8.5 h of treatment.

[a] Approximate nominal concentration values are presented, since Pluronic L121 was insoluble in FSW, forming white milky-like suspension.

[b] Morphological effects were concentration-related and independent of room temperature within the interval of 21–25 °C. Duplicate measurements showed no differences in NOEC, MEC, and MLC values.
